# Safety Assessment of Compounds after In Vitro Metabolic Conversion Using Zebrafish Eleuthero Embryos

**DOI:** 10.3390/ijms20071712

**Published:** 2019-04-06

**Authors:** Arianna Giusti, Xuan-Bac Nguyen, Stanislav Kislyuk, Mélanie Mignot, Cecilia Ranieri, Johan Nicolaï, Marlies Oorts, Xiao Wu, Pieter Annaert, Noémie De Croze, Marc Léonard, Annelii Ny, Deirdre Cabooter, Peter de Witte

**Affiliations:** 1 Laboratory for Molecular Biodiscovery, Department of Pharmaceutical and Pharmacological Sciences, University of Leuven, O & N II Herestraat 49-box 824, 3000 Leuven, Belgium; arianna.giusti@kuleuven.be (A.G.); xuanbac.hup@gmail.com (X.-B.N.); ceci.ranieri@gmail.com (C.R.); 18817595049@163.com (X.W.); annelii.ny@kuleuven.be (A.N.); 2 Pharmaceutical Analysis, Department of Pharmaceutical and Pharmacological Sciences, University of Leuven, O & N II Herestraat 49-box 923, 3000 Leuven, Belgium; stanislav.kislyuk@gmail.com (S.K.); melanie.mignot@kuleuven.be (M.M.); 3 Drug Delivery and Disposition, Department of Pharmaceutical and Pharmacological Sciences, University of Leuven, O & N II Herestraat 49-box 921, 3000 Leuven, Belgium; Johan.Nicolai@ucb.com (J.N.); marliesoorts@gmail.com (M.O.); pieter.annaert@kuleuven.be (P.A.); 4 L’Oréal Research & Innovation, 93600 Aulnay-sous-Bois, France; noemie.decroze@rd.loreal.com (N.D.C.); mleonard@rd.loreal.com (M.L.)

**Keywords:** drug discovery, toxicity, microsomes, metabolism, zebrafish eleuthero embryos

## Abstract

Zebrafish-based platforms have recently emerged as a useful tool for toxicity testing as they combine the advantages of in vitro and in vivo methodologies. Nevertheless, the capacity to metabolically convert xenobiotics by zebrafish eleuthero embryos is supposedly low. To circumvent this concern, a comprehensive methodology was developed wherein test compounds (i.e., parathion, malathion and chloramphenicol) were first exposed in vitro to rat liver microsomes (RLM) for 1 h at 37 °C. After adding methanol, the mixture was ultrasonicated, placed for 2 h at −20 °C, centrifuged and the supernatant evaporated. The pellet was resuspended in water for the quantification of the metabolic conversion and the detection of the presence of metabolites using ultra high performance liquid chromatography-Ultraviolet-Mass (UHPLC-UV-MS). Next, three days post fertilization (dpf) zebrafish eleuthero embryos were exposed to the metabolic mix diluted in Danieau’s medium for 48 h at 28 °C, followed by a stereomicroscopic examination of the adverse effects induced, if any. The novelty of our method relies in the possibility to quantify the rate of the in vitro metabolism of the parent compound and to co-incubate three dpf larvae and the diluted metabolic mix for 48 h without inducing major toxic effects. The results for parathion show an improved predictivity of the toxic potential of the compound.

## 1. Introduction

The process of drug discovery and development is characterized by several major steps including preclinical toxicity testing. Currently, despite the combination of numerous in vitro and animal models, the toxicity of a subset of compounds still remains undetected [1]. In many cases, this situation has led to a withdrawal of compounds from the market, to loss of confidence in medicines by the public and major financial implications for the companies affected [2]. Hence, there is a sense of urgency to explore new approaches for identifying compounds with a potential toxic profile in the early stages of drug discovery and development (DDD) [1].

As numerous studies have established the applicability of zebrafish in drug-toxicity screening [3,4,5,6,7,8,9,10,11], zebrafish-based model platforms have gained attention from the pharmaceutical industry as a valid way to evaluate novel drug candidates for their efficacy and safety [12]. First, zebrafish eleuthero embryos clearly show key advantages as an in vivo platform to identify safety liabilities [13]. For instance, a vertebrate zebrafish has a high degree of genetic conservation with humans. Approximately 70% of human genes have at least one zebrafish orthologue [14] and most of the important signal transduction pathways are preserved between zebrafish and humans [15]. Second, zebrafish-based toxicity models are cost-effective due to the high fecundity, rapid embryonic development, and easy maintenance of the animals [16,17]. Third, the eleuthero embryos are small so they can be arrayed in microtiter plates and exposed to compounds. As a consequence, only small quantities of the chemicals are needed which is of great importance in early DDD stages. However, in the framework of safety testing of compounds, zebrafish eleuthero embryos also come with some drawbacks. For instance, the capacity to metabolically convert xenobiotics is supposedly low and hence the effect of toxic metabolites formed in humans can easily go unobserved in zebrafish [18]. 

In order to improve the reliability of the zebrafish model, Busquet et al. developed the mDarT assay (subsequently also used by Weigt et al.) to assess the adverse effect of compounds modified upon metabolism [19,20]. To that end, the authors co-incubated two hpf zebrafish embryos with the compound of interest, in combination with rat liver microsomes (RLM). As these microsomes are a rich source of membrane phase I enzymes like CYPs, flavine-containing monooxygenases (FMO), esterases, amidases, and epoxide hydrolases, the embryos become in situ exposed to the metabolites of the compound generated continuously over time. However, the co-incubation of zebrafish embryos and RLM was limited to maximum 1 h due to the inherent toxicity of activated microsomes exerted on the embryos. Subsequently, Mattson et al. [21] modified the assay and showed that by exposing zebrafish embryos to a metabolic activation system in a later stage of their development, i.e., between 24 and 28 h post fertilization (hpf), the incubation period could be prolonged up to 4 h without causing developmental abnormalities in control conditions. 

Here we describe a novel comprehensive procedure exposing in a first step test compounds in vitro to RLM for 1 h at 37 °C. After a methanol precipitation step and evaporation of the supernatant, the reconstituted pellet containing the metabolic mix is analysed with ultra high performance liquid chromatography-Ultraviolet-Mass (UHPLC-UV-MS) to determine the disappearance rate of the parent compound due to metabolism, and to identify the metabolites formed. We as well as others, [22,23,24,25,26] are particularly interested in the toxic potential of compounds in later stages of embryonal development, i.e., when most organs have been formed, we then exposed zebrafish eleuthero embryos at three days post fertilization (dpf) to the metabolic mix. Using our protocol, a long incubation time of 48 h was possible (up to five dpf) without inducing major defects in control conditions.

To validate our protocol we used compounds undergoing different metabolic pathways: Parathion, an organophosphate insecticide activated to paraoxon through the action of CYPs enzymes [27,28] and malathion, another organophosphate insecticide that is rapidly converted by carboxylesterases to malathion monocarboxylic acid (MMC), a metabolic pathway competing with the formation of toxic malaoxon by CYPs [29,30]. The antibiotic chloramphenicol was used as a control compound, since its metabolic pathway relies mainly on glucuronidation, a phase II reaction not performed by microsomal enzymes [31]. 

## 2. Results

### 2.1. Adverse Effects of Test Compounds Unexposed to RLM in Zebrafish Eleuthero Embryos

First, the adverse effects of three test compounds, i.e., parathion, malathion and chloramphenicol, were determined without previous exposure to RLM, in zebrafish eleuthero embryos. To reproduce medium conditions that were identical to the ones used to incubate zebrafish eleuthero embryos with RLM-exposed test compounds (see further), blank samples containing RLM and microsome incubation buffer (MIB) were prepared and incubated at 37 °C, diluted with methanol and centrifuged. After evaporation of the supernatant, reconstitution of the residue and a further 4 fold-dilution in Danieau’s medium, the medium was spiked with different concentrations of each of the test compounds. In a next step, zebrafish eleuthero embryos (three dpf) were incubated with the compounds present in the so-obtained medium for 48 h, morphologically screened, and a mean score for the sub-lethal toxicity and lethality was assessed (see Table 1). Control conditions consisted of eleuthero embryos exposed to the RLM extract (without compound spiking) in the medium, and eleuthero embryos exposed to the medium without the RLM extract and without spiking. 

**Figure 1:**
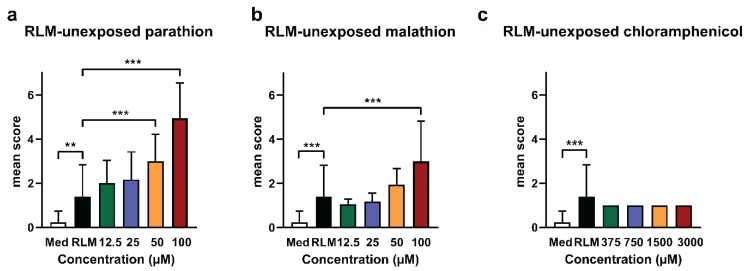
Mean scores of lethality and sub-lethal toxicity of test compounds unexposed to rat liver microsome (RLM) in zebrafish eleuthero embryos. The bar charts show the results after incubation of three days post fertilization (dpf) zebrafish eleuthero embryos with blank samples that were processed and spiked with different concentrations of (**a**) parathion, (**b**) malathion, and (**c**) chloramphenicol. Control conditions consisted of eleuthero embryos exposed to the RLM extract (without compound spiking) in the medium (indicated as RLM), and eleuthero embryos exposed to the medium without the RLM extract and without spiking (indicated as Med). After 48 h the incubated eleuthero embryos were morphologically screened, and the mean scores calculated, as described in methods. Three independent experiments were performed, the data were pooled and the mean ± SD was calculated. Hence, a total of six eleuthero embryos were processed per concentration, except in the case of Med samples (*n* = 10) and RLM samples (*n* = 10). For the statistical analysis, the mean score of RLM was compared with the mean scores of the other samples by using one-way ANOVA with Dunnett’s multiple comparison test. ** *p* ≤ 0.01, *** *p* ≤ 0.001.

Results are shown in Figure 1. Parathion and malathion elicited adverse effects in a dose-dependent way, whereas chloramphenicol did not. Compared to medium conditions, control conditions exposing eleuthero embryos to medium containing the RLM extract elicited a significantly higher mean score [0.23 (± 0.5) and 1.40 (± 1.43), respectively]. However, the sub-lethal toxic effects were mild in the latter conditions, as they consisted mainly of non-inflated swim bladders and sometimes a reduced touch response. Nevertheless, the data show that even after a methanol precipitation and a 4-fold dilution of the reconstituted pellet in the medium, some harmful effects of the microsomes remain. It should be pointed out however that these limited effects did not prevent the assessment of increased toxicity observed after the metabolic conversion of compounds, as in the case of parathion (see further). 

### 2.2. Quantitative Determination of Recovery Yield and Metabolic Conversion of Test Compounds after RLM-Exposure

Next, we determined the potential metabolic conversion of the test compounds by exposing the compounds to RLM for 1 h at 37 °C. The concentrations chosen for the test compounds were the ones eliciting an intermediate mean score of lethality and sub-lethal toxicity in the previous experiments, i.e., 200 µM, 200 µM, and 3 mM, in the case of parathion, malathion and chloramphenicol, respectively, taking into account that a final 4-fold dilution of the samples was necessary for the evaluation of the adverse effects of the RLM-exposed test compounds (see further).

The exposure was performed in the presence of the cofactors NADPH and G6P, known to activate specific metabolic enzymes present in RLM. In parallel, non-activated samples (without NADPH/G6P) were processed. Afterwards, samples were diluted with methanol, centrifuged and the supernatant was evaporated. Next, the dry extracts were resuspended in MilliQ water for analysis using UHPLC-UV-MS and the metabolites formed were identified based on their retention time and m/z ratio (see Figure 3 for an overview of the workflow). 

The recovery of parathion with regards to the initial concentration was 55.8% ± 11.6 (mean ± SD) (*n* = 3) in the non-activated sample and 40.5 % ± 15.8 in the activated one, showing a metabolic conversion ca of 15%. Further analysis confirmed the formation of paraoxon in the activated sample, as demonstrated by the presence of an ion with an m/z ratio of 276, in addition to the presence of parathion (m/z ratio: 292) (Supplementary Figure S1). Other metabolites were not detected. Unfortunately, the amount of paraoxon formed could not be quantified as the compound can pose an extreme health risk under standard safety measures used in the laboratory.

The amount of malathion in both activated and non-activated samples was non-quantifiable (recovery < 3%) (*n* = 3). Instead, in both samples we found a compound present that was tentatively identified as monocarboxylic acid derivatives of malathion (MMC) (Supplementary Figure S2), suggesting that malathion was entirely converted to MMC irrespective of the conditions (activation or not). The amount of MMC formed was not quantified, as the compound is commercially not readily available.

The recovery of chloramphenicol amounted to 86.3 % ± 2.9 and 87.7 % ± 0.6 (mean ± SD) (*n* = 3) in the non-activated and activated samples, respectively. This result, together with the lack of detection of any metabolite (Supplementary Figure S3), suggests the absence of any metabolic conversion of the compound.

### 2.3. Adverse Effects of Test Compounds Exposed to RLM in Zebrafish Eleuthero Embryos

We then investigated the adverse effects of test compounds exposed to RLM (activated or not) in zebrafish eleuthero embryos. The samples used for the analysis with UHPLC-UV-MS (see above) were first diluted 4-fold in Danieau’s medium. This was a necessary step as MIB contains a high amount of salt not completely removed by the methanol precipitation step, but also because the reconstituted and undiluted extracts of the microsomes were lethal for zebrafish eleuthero embryos. 

**Figure 2:**
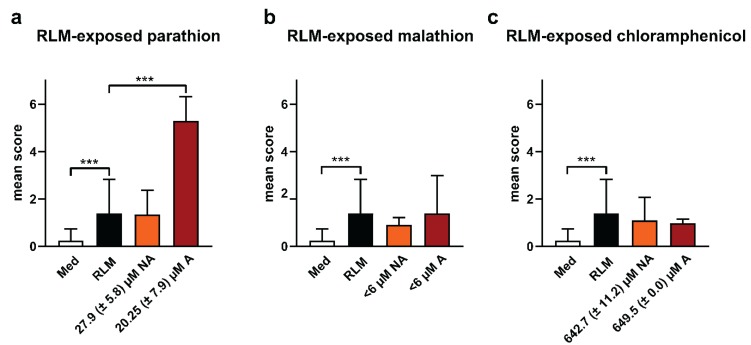
Mean scores of lethality and sub-lethal toxicity of test compounds (**a**) parathion, (**b**) malathion, and (**c**) chloramphenicol previously exposed to RLM activated (A) or not (NA) with reduced β-nicotinamide adenine dinucleotide 2′-phosphate (NADPH) and glucose-6-phosphate (G6P) in zebrafish eleuthero embryos. The bar charts show the results after incubation of three dpf zebrafish eleuthero embryos with 4-fold dilutions of reconstituted extracts of processed samples that were analyzed on their content (see Figure 3). Control conditions consisted out of eleuthero embryos exposed to the RLM extract (without compound spiking) in the medium (indicated as RLM), and eleuthero embryos exposed to the medium without the RLM extract and without spiking (indicated as Med). After 48 h the incubated eleuthero embryos were morphologically screened, and the mean scores calculated, as described in methods. Three independent experiments were performed, the data were pooled and the mean ± SD was calculated. Hence, a total of 30 eleuthero embryos were processed per condition. For the statistical analysis, the mean score of RLM was compared with the mean scores of the other samples by using one-way ANOVA with Dunnett’s multiple comparison test. *** *p* ≤ 0.001.

The results show that in the case of non-activated parathion, the compound evoked adverse effects at a calculated concentration of 27.9 ± 5.8 µM (taking into account the recovery and dilution step) that was somewhat comparable with blank samples spiked with similar concentrations of parathion (Figure 1a and Figure 2a). Conversely, parathion exposed to activated microsomes induced a mean score of lethality and sub-lethal toxicity (at a calculated concentration of 20.25 ± 7.9 µM) that was substantially higher than the corresponding spiked blank samples (Figure 1a and Figure 2a), implying that one metabolite (i.e., paraoxon) was formed that exhibited a considerable higher toxicity than the parent compound. 

As mentioned before, malathion could be recovered only in small amounts from the samples exposed to RLM (calculated conc < 6 µM). However, the presence of an ion with an m/z ratio of 302 in both the activated and non-activated samples suggests that malathion was metabolized to a great extent to MMC in a cofactor-independent way. Since the incubation of zebrafish eleuthero embryos with the activated and non-activated samples obtained after a 4-fold dilution in Danieau’s medium did not elicit an increase in the mean score of toxicity (compared to control), the results show that the metabolite formed demonstrated a somewhat similar safety profile as the parent compound (Figure 2b).

Chloramphenicol elicited adverse effects that were identical in both the non-activated and activated samples [calculated concentrations: 642.7 (± 11.2) µM and 649.5 (± 0.0) µM, respectively] (Figure 2c). Altogether, the data are in line with the finding that the compound was not metabolized in the conditions used. 

## 3. Discussion

Zebrafish have been proposed as a valid in vivo model for studying acute and chronic safety issues of chemicals. To date, over thousands of studies have been published since the initial paper (1965) that explored the effect of zinc sulphate during the different stages of zebrafish development [32]. Many of these studies have shown a high concordance of compound toxicity between zebrafish and humans [33,34]. Nevertheless, it is uncertain whether zebrafish eleuthero embryos have a sufficient capacity to metabolically convert xenobiotics in an adequate way, and little is known regarding the identity of the metabolites formed [18,35]. As a matter of fact, it was shown very recently that the activity of proteins involved in the metabolism of xenobiotics are generally low to undetectable before 72 h post-fertilization [36]. Therefore, since metabolism might increase or reduce the toxic profile of compounds dramatically, the use of eleuthero embryos might result in false negative or positive results.

In an effort to circumvent this concern, we developed a comprehensive methodology exposing in a first step compounds in vitro to RLM for 1 h at 37 °C, followed by an analysis of the metabolic conversion and metabolites present in the sample extracts. In the next step, three dpf zebrafish eleuthero embryos were exposed for 48 h at 28 °C to the metabolic mix diluted in Danieau’s medium, followed by a stereomicroscopic examination of adverse effects. We used three compounds, i.e., parathion, malathion and chloramphenicol, to demonstrate the potential of our methodology.

We first incubated the eleuthero embryos from three to five dpf with reconstituted and diluted extracts of blank samples spiked with different concentrations of the test compounds in order to determine their intrinsic toxicity. After selecting concentrations that caused signs of intermediate sub-lethal toxicity, compounds were incubated with activated and non-activated RLM and the adverse effects tested.

Parathion is an organophosphate insecticide that is highly toxic to mammals by all routes of exposure (e.g., the oral LD50 in rat is 2 to 30 mg/kg) [37]. Of interest, after absorption the compound is rapidly converted into paraoxon, the active anticholinesterase metabolite that causes poisoning [38]. In humans the CYPs enzymes responsible for the conversion of parathion to paraoxon are CYP2B and CYP3A [27,28]. Although those isoforms of the enzymes are different in rats [39], they metabolize parathion in the same way as humans, thus providing the toxic metabolite paraoxon. When zebrafish eleuthero embryos were incubated with parathion (without exposure to RLM) with concentrations up to 50 µM, the adverse effects were dose-related but still much lower than anticipated from a highly toxic and lipophilic compound that is likely taken up readily by eleuthero embryos [40]. Conversely, in the case of microsomally-activated parathion, a pronounced toxic and lethal effect was obtained upon generation of paraoxon. These results therefore seem to indicate that parathion is only limitedly or not activated by eleuthero embryos, leading to a false negative outcome when the compound is not pre-metabolized. 

Malathion is a relatively safe organophosphate insecticide, with an oral LD50 in a rat of 1350 mg/kg [41]. Its rapid degradation by carboxylesterases competes with the cytochrome P450-catalyzed formation of malaoxon, the toxic metabolite [30]. The production of malaoxon relates to different enzymes that are involved in the metabolic generation of the compound depending on the concentration of malathion present: CYP1A2 is mainly involved at low malathion concentrations, whereas CYP2B6 and 3A4 have a more prominent role at high µmolar concentrations [30]. CYP1A2 has a strong conservation among species, with an identity between humans and rats of 83 and 80% for CYP1A1 and CYP1A2, respectively [42]. In contrast CYP2B and 3A show different isoforms and substrate specificity among species. In our experimental setup, malaoxon was not generated in detectable amounts, whereas MMC, a monocarboxylic acid derivative of malathion, was found in both the activated and non-activated samples. MMC is the major metabolite of malathion formed as a result of the activity of carboxylesterases. Of interest, the latter enzymes do not need cofactors for their activation. The outcome therefore suggests that a high carboxylesterase activity led to a complete decarboxylation of the parent compound. As a result, no malaoxon was produced, and compared to the results obtained for malathion, no increased toxicity was observed. This is in line with the fact that malathion is a relative safe chemical that is used as an insecticide in public health pest control programs, as well as in household products like lotions to treat head lice in adults and children [43,44]. 

Chloramphenicol is a bacteriostatic man-made antibiotic with a broad-spectrum activity and a low incidence of toxicity. The compound is mainly eliminated by the human liver after *O*-glucuronidation [45]. Chloramphenicol has also been found to retard the metabolic transformation of a variety of drugs such as the tolbutamide, diphenylhydantoin and dicoumarol in man and the hexobarbital in mice by acting on some CYP drug-oxidizing enzymes [46]. Moreover, it was demonstrated that the activation of chloramphenicol by RLM involves a *p*-450 monooxygenase but only after induction of the enzyme by phenobarbital treatment [47]. It is therefore reasonable to state that the high concentration of chloramphenicol used (i.e., 3 mM) resulting in an inhibition of CYP drug-oxidizing enzymes, together with the fact that the rats were not pre-treated with phenobarbital, gave rise to a complete lack of metabolism. As a result, no differences in adverse effects were observed for all conditions tested. 

Taken together, we successfully developed a platform combining the high-throughput of an in vivo zebrafish-based toxicity test with a mammalian pre-metabolism step. We were able to show that parathion is converted metabolically into paraoxon, an activation step that is needed to confirm its well-known high toxicity in eleuthero embryos. Conversely, malathion that is rapidly metabolized into a decarboxylation product, thereby outcompeting the production of the toxic malaoxon, as well as chloramphenicol that is known to be only very limitedly converted in phase I-metabolites, did not show levels of adverse effects that were different between the parent and RLM-exposed compounds. Although validating the platform with many more compounds is necessary to further confirm its applicability, we believe that the comprehensive methodology developed herein is promising in identifying compounds that are converted into toxic/non-toxic metabolites via phase I metabolism, thereby increasing the possibility to detect harmful chemicals in an early drug discovery phase.

## 4. Materials and Methods

### 4.1. Chemicals

Parathion, malathion, chloramphenicol, dimethyl sulfoxide (DMSO), ethylenediaminetetraacetic acid (EDTA), sucrose, NaH_2_PO_4_.H_2_O, NaH_2_PO_4_.2H_2_O, and MgCl_2_.6 H_2_O were purchased from Sigma-Aldrich (St. Louis, MO, USA). The reduced β-nicotinamide adenine dinucleotide 2′-phosphate (NADPH) and glucose-6-phosphate (G6P) were obtained from Hoffmann la Roche (Basel, Switzerland). Xylazine 2% was from V.M.D. (Arendonk, Belgium). Ketamine HCl 115 mg/mL was purchased from Eurovet (Heusden-Zolder, Belgium). The BCA assay kit was obtained from Thermo Fisher Scientific (Asse, Belgium), the formic acid (MS-grade) from Acros Organics (Geel, Belgium), acetonitrile (ACN) (analytical grade) from Fischer Scientific (Loughborough, UK) and 3-morpholinopropane-1-sulfonic acid (MOPS) from the MSD research laboratory (Rahway, NJ, USA). Test compounds were dissolved in DMSO to obtain stock solutions (parathion: 50 mM, malathion: 50 mM, chloramphenicol: 750 mM) that were stored at −20 °C.

### 4.2. Fish Eleuthero Embryo Tests

All experiments carried out were approved by the Ethics Committee of the University of Leuven (number P154/2015, approval date 1 October 2015) and by the Belgian Federal Department of Public Health, Food Safety & Environment (license number LA1210261). Zebrafish were maintained in a UV-sterilized rack recirculating system equipped with a mechanical and biological filtration unit and kept under a 14/10  h light/dark cycle at the temperature of 27–28  °C and pH of 6.8–7.5. Water quality was monitored daily for pH, temperature and conductivity, and weekly for ammonia and nitrite levels (SL1000 Portable Parallel Analyzer, Hach Instruments, Loveland, CO, USA) and nitrate (Tetra, Melle, Germany). Zebrafish were fed three times per day, twice with flake food (TetraMin, Tetra, Melle, Germany) and once with Artemia (brine shrimp). Embryos were obtained via natural group spawning (ratio males/females: about 1:1) over marbles, sorted and kept in a petri dish (92  ×  16 mm Sarstedt (Nümbrecht, Germany)) at 28  °C in a Peltier-cooled incubator (IPP 260, Memmert, Schwabach, Germany) in Danieau’s solution (1.5  mM HEPES, 17.4  mM NaCl, 0.21  mM KCl, 0.12  mM MgSO4, and 0.18 mM Ca(NO3)2 and 0.6  μM methylene blue) with a density of 50 embryos per 50  mL. 

Fertilized eggs of good quality (fertilized, clear cytoplasm and symmetric cleavage), collected from wild type AB zebrafish genitors, were selected for experiments and kept in petri dishes containing Danieau’s solution until the time of compound exposure. All eleuthero embryos were derived from the same spawns of eggs for the comparison between the control and treated groups. Mortality in untreated groups of embryos was <10%.

### 4.3. Determination of Sub-Lethal Toxicity and Lethality of Test Compounds Exposed or Unexposed to RLM in Zebrafish Eleuthero Embryos

**Figure 3:**
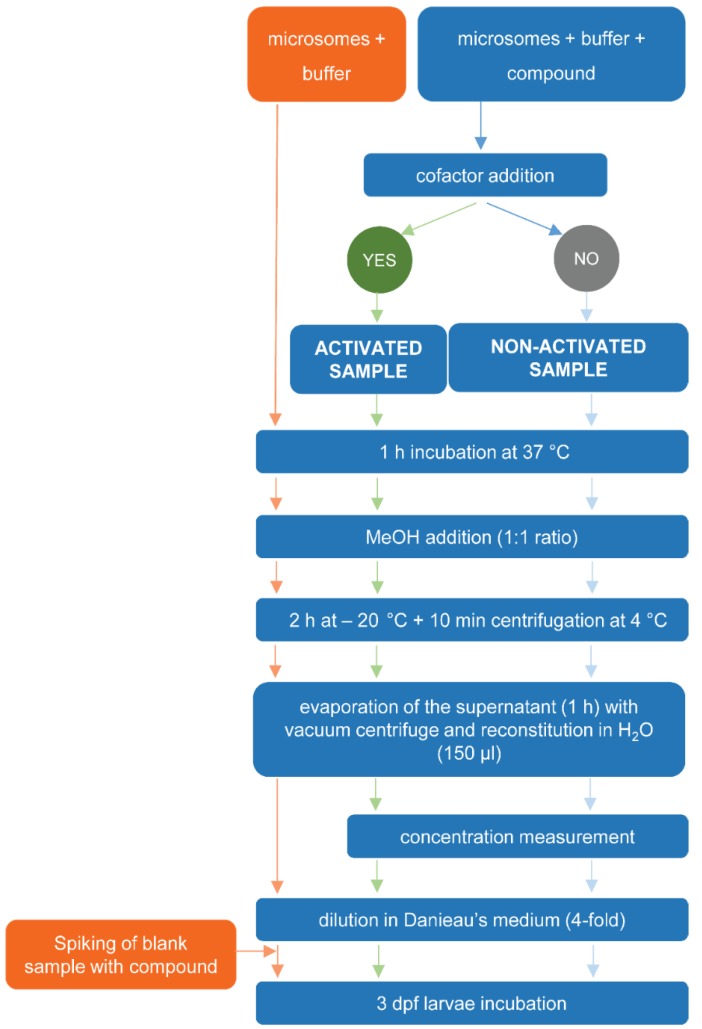
Workflow for the determination of sub-lethal toxicity and lethality of test compounds exposed or unexposed to RLM in zebrafish eleuthero embryos.

A workflow of the different steps is depicted in Figure 3.

#### 4.3.1. Exposure of Test Compound or Blank Samples to RLM

RLM prepared as described before [48] and stored at –80 °C in 0.5 mL aliquots were thawed and kept on ice. Subsequently, they were diluted with microsomal incubation buffer (MIB) (3 mM MgCl_2_, 100 mM sodium phosphate buffer pH 7.4) to 2 mg/mL microsomal protein. Solutions (150 µL) of test compounds were made freshly in MIB and pre-exposed to RLM (75 μL) for 5 min at 37 °C. Then, 75 μL of pre-warmed mixture of NADPH (4 mM) and G6P (12 mM) in MIB was added to the vials to start the metabolic activation. The final concentrations of the compounds for the exposure to microsomes were 200 µM, 200 µM and 3 mM for parathion, malathion and chloramphenicol, respectively. In the case of non-activated samples, 75 μL of pre-warmed MIB (without NADPH/G6P) was added. In the case of blank samples, MIB (225 µL) was supplemented to RLM (75 µL) and no test compound or NADPH/G6P was added. The in vitro exposure of test compound or blank samples to RLM was carried out in clear glass vials at 37 °C using an IKA KS 4000 i control shaking incubator (IKA, Staufen, Germany) set at 200 rpm. After 1 h methanol (300 µL) was added to the test compound or blank samples.

#### 4.3.2. Preparation of Sample Extracts

Next, samples were ultrasonicated (Diagenode Bioruptor Plus, Seraing, Belgium) at 4 °C. The overall treatment time was 10 min spread over 10 cycles of 30 s with pauses of 30 s in-between. Subsequently, samples were placed at –20 °C for 2 h and centrifuged at 4 °C for 10 min at 10,000 × *g* (Centrifuge 5424 R, Eppendorf, Hamburg, Germany) to remove excess of phosphate buffer and microsomal debris. Next, the supernatant was split over 3 × 1.5 mL Eppendorf tubes (approximately 190 μL for each tube) and centrifuged with a vacuum concentrator connected to an oil pump for 1 h at RT in V-AQ mode (concentrator plus, Eppendorf, Hamburg, Germany).

#### 4.3.3. Quantitative Determination of Recovery Yield and Metabolic Conversion of Test Compounds Exposed to RLM

In the case of the test compounds exposed to RLM (activated and non-activated samples), the obtained dry extracts were resuspended in 150 µL of MilliQ water for analysis (pooling each sample split over three Eppendorf tubes for evaporation, in 150 µL). Analyses were performed using an Agilent Infinity 1290 UHPLC system (Agilent, Waldbronn, Germany) consisting of an autosampler, quaternary pump and DAD-detector in combination with a Bruker Esquire 3000 plus iontrap mass spectrometer (Bruker, Bremen, Germany) with an electrospray ionization (ESI) source. The capillary voltage was set at + 4kV, with a drying gas flow rate of 8 L/min and a drying temperature of 365 °C. The nebulizer gas pressure was set at 45 psi. All samples were analyzed in a positive ion mode. The scan rate was set to 120–1000 m/z. Data acquisition and peak processing were performed using the OpenLAB CDS Chemstation Edition 01.04 software (Agilent, Waldbronn, Germany) and Compass 1.3 EsquireControl Version 6.2 (Bruker Daltonik, Billerica, MA, USA). All calculations concerning the evaluation of the recorded data were made in MS Excel (Microsoft Corporation, Seattle, USA). The compounds were separated on an Acquity BEH C18 column (100 mm–2.1 mm, dp = 1.7 µm) from Waters (Milford, MA, USA) at a flow rate of 0.4 mL/min. Gradient elution was performed starting at 97:3 (*v*/*v*) 10 mM ammonium formate (pH 2.8 modified with formic acid):ACN, and changed to 18:82 (*v*/*v*) 10 mM ammonium formate (pH 2.8):ACN in 10.5 min. After elution of the compounds, a cleaning step using 100% ACN was applied for 1.3 min after which the column was re-equilibrated for 7.0 min at initial conditions. The injection volume was 1 µL. Chloramphenicol and parathion were detected and quantified at 254 nm, malathion by extracting the ion chromatogram at m/z = 331. The metabolites of the compounds were tentatively identified based on their observed m/z. For the quantification of the compounds, calibration curves were built by spiking blank microsome samples (obtained after sample preparation) at minimum five different levels (between three and 100 µM for parathion, between six and 100 µM for malathion and between 47 and 3000 µM for chloramphenicol). Each concentration was injected five times and the variability (calculated as the relative standard deviation %RSD, *n* = 5) was between 0.5 and 9.0% for parathion, between 1.2% and 6.7% for malathion and between 0.2% and 0.4% for chloramphenicol. Linear curves with R2 ≥ 0.993, R2 ≥ 0.981 and R2 ≥ 0.999 were obtained for parathion, malathion and chloramphenicol, respectively. 

Compound recovery was calculated by dividing the concentration found in the sample by the initial concentration, and then multiplied by 100 to yield a percentage. Metabolic conversion was determined by subtracting the concentration found in the activated sample from the one present in the non-activated condition.

#### 4.3.4. Biological Assay Using Zebrafish Eleuthero Embryos

The eleuthero embryos were arrayed in 96-well plates (Falcon^®^, Corning, Lasne, Belgium) (1 larva/well, 100 µL/well) and incubated with the test compounds exposed (activated and non-activated samples) (*n* = 10) or unexposed to RLM (*n* = 6) for 48 h in an incubator (28 °C, 14/10-h light/dark cycle). Control conditions consisted of eleuthero embryos exposed to the RLM extract (without compound spiking) in the medium (*n* = 10), and eleuthero embryos exposed to the medium without the RLM extract (and without spiking) (*n* = 10). Data were pooled together from three independent experiments. 

In the case of determining the adverse effects of the test compounds unexposed to RLM, after processing the blank samples (containing only RLM in MIB as described), the dried extracts were reconstituted, diluted 4-fold in Danieau’s medium, and finally spiked with different concentrations of the individual compounds (12.5-100 µM in the case of parathion and malathion, and 375 µM-3000 µM in case of chloramphenicol). In the case of the test compound samples (at a selected concentration per compound) that were exposed to RLM (activated and non-activated), after reconstitution of the dried extracts and quantitative determination of the concentration using UHPLC-UV-MS (as described), samples were diluted 4-fold in Danieau’s medium. 

**Figure 4:**
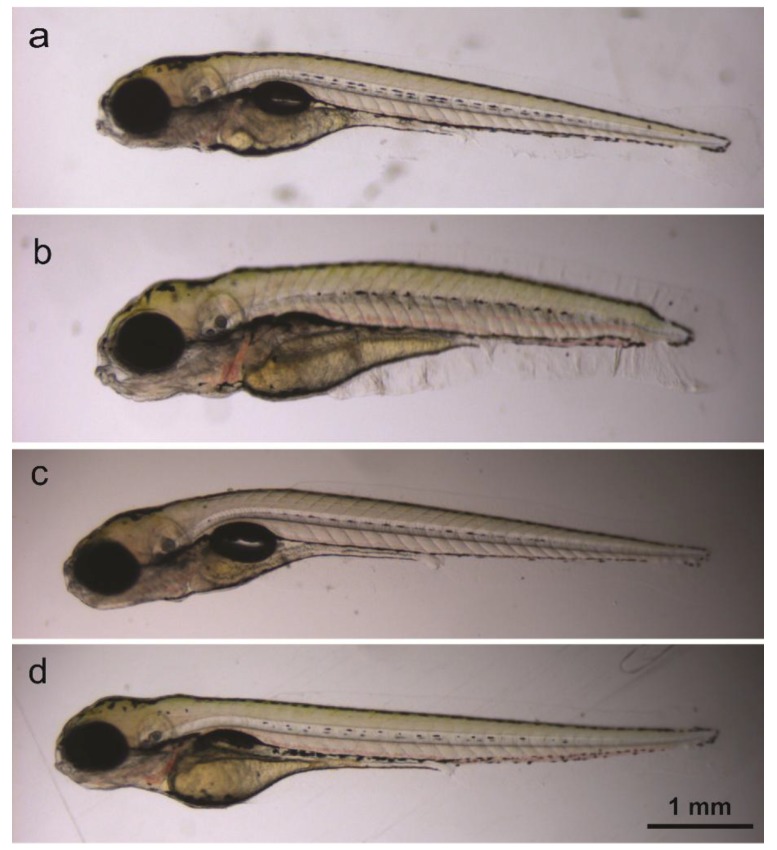
Lateral view of untreated five-dpf eleuthero-embryo (**a**), and of compound-treated eleuthero-embryo with abnormal body shape and non-inflated swim bladder (**b**), with curved body (**c**) and with non-inflated swim bladder (**d**).

At five dpf, the absence or presence of lethal effects (no heartbeat, degraded body) and touch response was assessed using non-anesthetized eleuthero embryos observed under a stereomicroscope (M80, Leica Microsystems, Germany). The absence or presence of morphological defects (see Table 1, Figure 4) was determined while eleuthero embryos were anesthetized in 0.5 mM tricaine. Pictures were taken with a DFC3 10 camera mounted on the stereomicroscope and stored.

A mean score of lethality and sub-lethal toxicity per condition was calculated as follows: To each adverse effect observed (see Table 1) a score of one was given (maximum four per embryo), and to each dead embryo a score of six was assigned. The mean score was then calculated for all eleuthero embryos (pooled results) examined per condition.

## 5. Conclusions

Using parathion, malathion and chloramphenicol as test compounds, we developed a new comprehensive procedure to in vitro metabolize chemicals using rat liver microsomes (RLM) prior to the exposure of zebrafish eleuthero embryos. The novelty of our method relies in the possibility to quantify the rate of metabolism of the parent compound after the incubation with RLM and to incubate three dpf larvae for 48 h with the diluted metabolic mix without inducing major toxic effects. In the case of parathion, the data show that the incubation of zebrafish eleuthero embryos with the in vitro metabolized chemical resulted in a substantially improved prediction of the toxic potential of the compound.

## Figures and Tables

**Table 1:** Description of sub-lethal toxic effects observed in zebrafish eleuthero embryos at 5 dpf.

Adverse Effect	Description
Bad development (BD)	Truncated body/ length and abnormal body shape (Figure 4a)
Curved body (CB)	(Figure 4b)
Impaired motility (IM)	No touch response or reduced touch response
Swim bladder defects (SBD)	Absent/undeveloped swim bladder (Figure 4a,c)

## Supplementary Materials

Supplementary materials can be found at https://www.mdpi.com/1422-0067/20/7/1712/s1.

## Abbreviations

RLM	Rat liver microsomes
UHPLC-UV-MS	Ultra high performance liquid chromatography-Ultraviolet-Mass
dpf	Days post fertilization
hpf	Hours post fertilization
mDarT assay	Danio rerio teratogenic assay with metabolic activation
MIB	Microsome incubation buffer
NADPH	Reduced β-nicotinamide adenine dinucleotide 2′-phosphate
G6P	Glucose-6-phosphate
MMC	Monocarboxylic acid derivatives of malathion
