# Supplementary material for: Safety Assessment of Compounds after In Vitro Metabolic Conversion Using Zebrafish Eleuthero Embryos
Source: Int J Mol Sci. 2019 Apr 6;20(7):1712. doi: 10.3390/ijms20071712 (PMC6479637; doi:10.3390/ijms20071712)

Supplementary Materials

Figure S1. Extracted ion chromatograms of non-activated (a) and activated sample (b) of parathion. Parathion is present in both the non-activated and activated samples with a mass-to-charge ratio (m/z) of 292 corresponding to the molecular weight of parathion (291), as verified with a reference standard. Paraoxon is present in the activated sample with an m/z = 276 corresponding to molecular weight of paraoxon (275) (mass spectra were recorded in positive ESI mode).

Non-activated sample

a

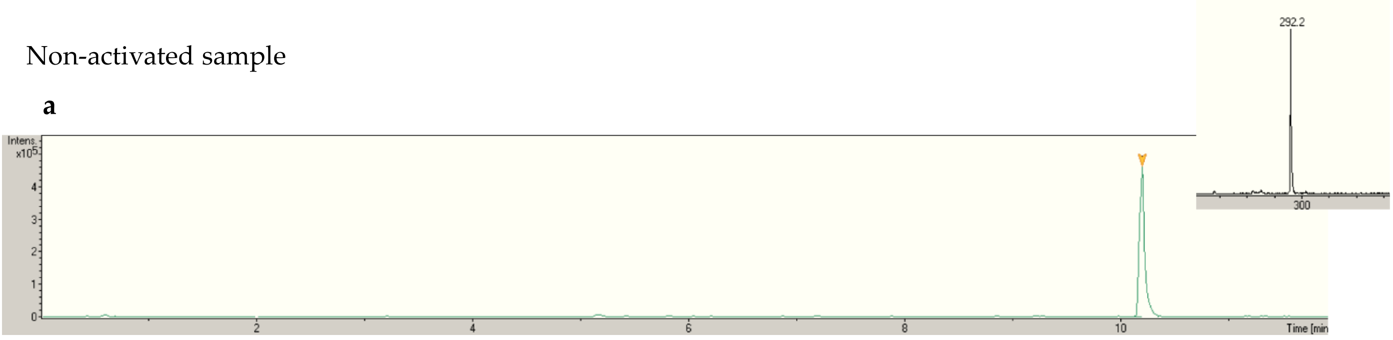

Activated sample

b

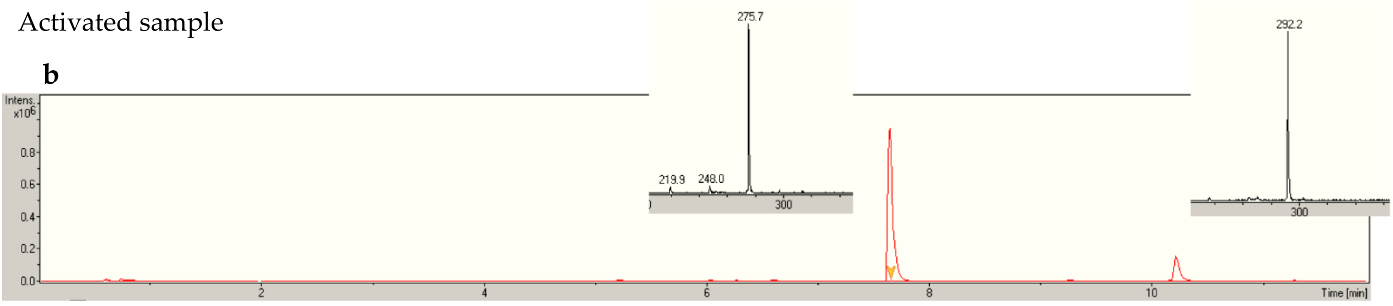

Figure S2. Extracted ion chromatograms of non-activated (a) and activated sample (b) of malathion. In both the non-activated and activated samples there is a peak with a mass-to-charge ratio (m/z) of 303 corresponding to the molecular weight of MMA (301) (mass spectra were recorded in positive ESI mode). No reference standard of MMA was available, so its identification is only tentative.

Non-activated sample

a

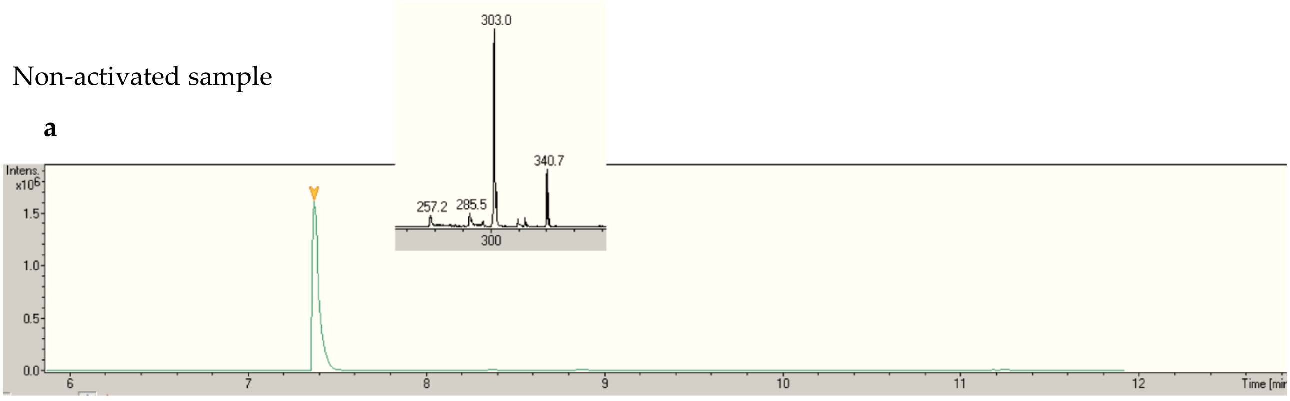

Activated sample

b

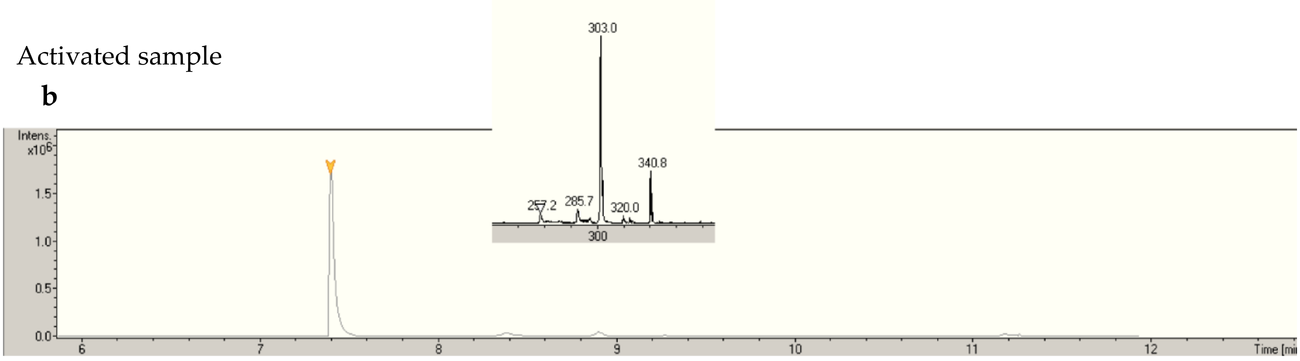

Figure S3. LC-UV chromatogram of non-activated (a) and activated sample (b) of chloramphenicol. In both the non-activated and activated samples there is a peak with an elution time of 5.6 corresponding to chloramphenicol (as verified with a reference standard).

Non-activated sample

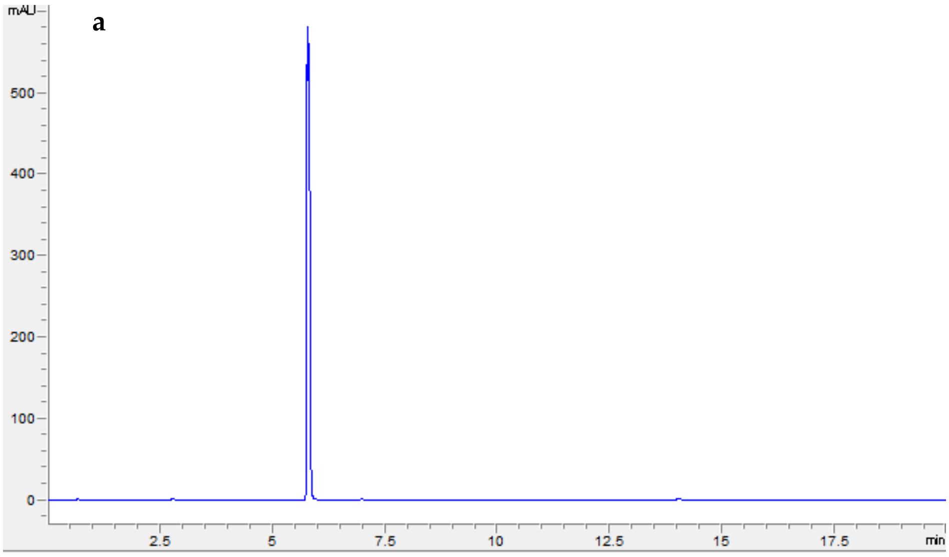

Activated sample

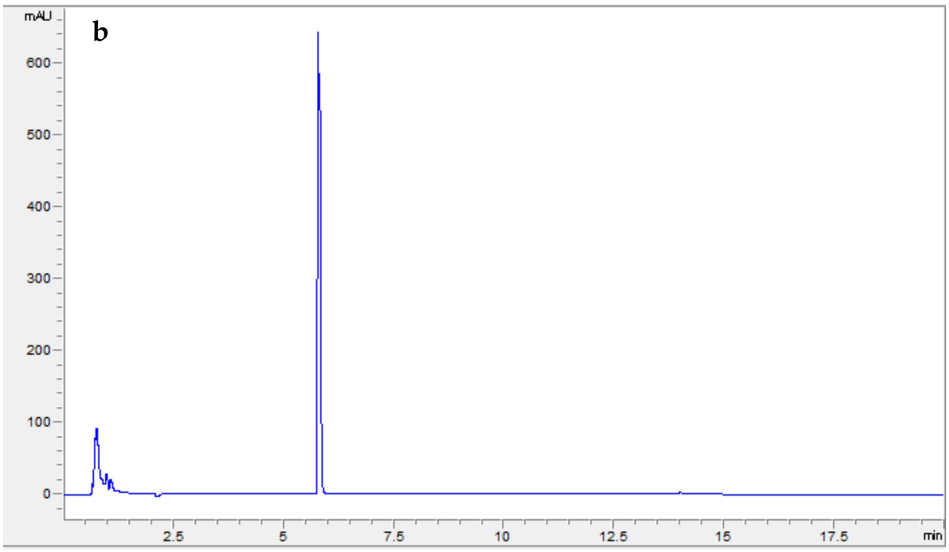

Supplement: Supplementary file 1 [file ijms-20-01712-s001.pdf]
